# Knockdown of ALPK2 inhibits the development and progression of Ovarian Cancer

**DOI:** 10.1186/s12935-020-01347-z

**Published:** 2020-06-24

**Authors:** Xiaogang Zhu, Siqi Yan, Songshu Xiao, Min Xue

**Affiliations:** 1 Department of Obstetrics and Gynecology, the Third Xiangya Hospital of Central South University, Changsha, 410013 Hunan China; 2 Department of radiation oncology, Hunan Academy of traditional Chinese medicine affiliated hospital, Changsha, Hunan 410007 China

**Keywords:** ALPK2, OC, Proliferation, Cell cycle, Apoptosis, Migration

## Abstract

**Background:**

Alpha protein kinase 2 (ALPK2) was known to play a vital role in cancer by regulating cell cycle and DNA repair. Ovarian cancer (OC) is one of the most lethal malignancies in the female reproductive system. The emphasis of this study is to explore the role of ALPK2 in OC.

**Methods:**

Firstly, tumor and normal tissues were collected for detecting expression of ALPK2 in OC. Lentivirus-mediated shRNA knockdown of ALPK2 was used to construct OC cell model, which was verified by qRT-PCR and Western blot. The cell proliferation was detected by MTT, cell cycle and apoptosis were measured through flow cytometry. Wound-healing assay was conducted to detect the migration of OC cells.

**Results:**

It was proved that the expression of ALPK2 in OC tissues was significantly higher than that in normal ovarian tissues. Moreover, knockdown of ALPK2 could inhibit proliferation, migration and promote apoptosis, arrested cell cycle of OC cells. It was also found that ALPK2 knockdown inhibited tumor growth in xenograft mice in vivo. Furthermore, ALPK2 was involved in OC cells via regulating EMT-related proteins (N-cadherin, Vimentin and Snail), inhibiting apoptosis-related proteins (Bcl-2, Bcl-w, HSP27, HSP60, IGF-I, IGF-1sR, Survivin and XIAP), as well as the regulation of downstream pathways (Akt, p-Akt, Cyclin D1, CDK6 and PIK3CA).

**Conclusions:**

In conclusion, ALPK2 might serve as an optional target for prognosis and therapeutic of OC patients.

## Introduction

Ovarian cancer (OC) is one of the most common malignant tumors of the female reproductive system, ranking third in incidence [1]. Based on histopathology and molecular genetics, there are five main types of OC: advanced serous (70%), endometrioid (10%), clear cell (10%), mucous (3%), and low serous < 5%) [2]. In addition, the heterogeneity of OC includes type I and type II. Type I is divided into three subtypes: those associated with endometriosis (endometrioid, clear cells, serous or mixed mullerian tumors), fallopian tubes (low-grade serous tumors) and germ cells or transitional cells (mucinous and Bolner’s tumors); while type II seems to originate from fallopian tube epithelium, originated from Müllerian cells [3]. This indicated that OC is a related but distinct family of tumors, with significant differences in molecular, clinicopathological and behavioral characteristics. A similar feature is that most OC patients are asymptomatic and more than half of them are diagnosed with advanced stage and suffered from extremely poor survival [4]. In recent years, with the application of paclitaxel combined with platinum-based chemotherapy, intraperitoneal chemotherapy and low-risk surgery, the 5-year survival rate is still lower than 40% [5, 6]. At present, changes in cell growth rates and the occurrence of malignant tumors are thought to be closely related to abnormal gene expression [7]. However, as polygenic disease, the biological behavior mechanism of OC induction is still unknown. Consequently, it is of great significance to study the molecular mechanisms for facilitating the inhibition of progression and metastasis of OC.

The member of an atypical alpha protein kinase family member, alpha protein kinase 2 (ALPK2) protein family has a highly conserved *α* protein kinase domain, which, unlike the traditional protein kinase, is evolutionarily limited to vertebrates [8]. *ALPK2* gene has a strong similarity with elongation factor 2 kinase catalytic domain, which is located at 18q21.31 [9]. In addition, ALPK2 has been reported to be essential in cancer by regulating cell cycle and DNA repair genes [10]. Indeed, Lawrence et al. recently reported *ALPK2* to be one of 33 genes whose somatic point mutations were associated with 21 cancer cell lines [11]. Recent comprehensive sequence analysis of potential oncogenic kinase mutations has shown that ALPK2 is one of the mutated genes in OC [12–15]. Up to now, no functional studies on ALPK2 in OC have been reported.

This study aimed to determine the function of ALPK2 in human OC. To this end, specific small interfering (si) RNA was transfected into various OC cells, and the effects of ALPK2 on cell proliferation, cycle distribution, apoptosis, and migration were studied through MTT, flow cytometry and Transwell, wound-healing. In general, ALPK2 knockdown could suppress the development and progression of OC, providing new insights for the diagnosis and treatment.

## Materials and methods

### Ovarian tissue microarray and cell culture

230 OCs and their marginal normal tissue chips were purchased from Xi’an Alina Biotechnology Co., Ltd., including 30 normal ovarian tissue chips and 200 OC tissue chips. The OC tissue chip included 134 cases of serous adenocarcinoma, 33 cases of mucinous adenocarcinoma, 2 cases of adenocarcinoma, 7 cases of endometrioid adenocarcinoma, 16 cases of metastatic carcinoma, and 8 cases of normal marginal ovarian tissue. Collection of samples with informed consent from patients.

The OC cell lines HO-8910 and OVCAR-3 were purchased from the Cell Bank of the Chinese Academy of Sciences (Shanghai, China). They were cultured in DMEM medium supplemented with 20% FBS, maintaining 5% CO_2_, 37 °C, and 75 ± 5% humidity air.

### Immunohistochemical staining (IHC)

The tissue sections were deparaffinized, repaired and blocked with citric acid antigen, they were incubated with ALPK2 antibody (1:100,Abcam, USA, Cat. # ab111909) at 4 °C overnight. After elution with PBS for 5 times, secondary antibody IgG (1: 400, Abcam, USA, Cat. # ab6721) was added and incubated at room temperature for 30 min. Tissue sections were subsequently stained with DAB and hematoxylin for visualization. Finally, images were taken under a microscope and evaluated according to the German immune response score [16]. In summary, the high or low expression level of ALPK2 in OC tissues is defined by the total score of positive cells and total staining intensity.

### Lentiviral shRNA vector construction and cell transfection

The RNA interference target sequence (5′-GCGAAGACCTTGGCATTTATT-3′) was designed based on the RNA interference sequence design principle for ALPK2. Lentiviral shRNA vector targeting ALPK2 was constructed by Shanghai bioscienceres Co. Ltd. (Shanghai, China). The sequences targeting of ALPK2 were synthesized and cloned into a lentiviral vector BRV-108 (Shanghai Yibeirui bioscienceres Co. Ltd., Shanghai, China). Monoclonal clones on the plate were selected for PCR identification, and the positive clones were sequenced and analyzed. The positive clones were cultured and extracted to obtain high purity plasmids (EndoFree midi Plasmid Kit, TIANGEN, Cat. #DP118-2) for downstream virus packaging. 293T cells were co-transfected with three plasmids (BRV-108, Helper 1.0 and Helper 2.0) to obtain lentivirus. The negative control group was transfected with negative lentivirus shCtrl, and the shALPK2 group was transfected with shALPK2. After 72 h, the expression of green fluorescent protein was observed with a fluorescence microscope to evaluate the transfection efficiency.

### Quantitative real-time -PCR

Total RNA was extracted from cell samples according to the kit instructions (Invitrogen, Carlsbad, CA, USA). The Reverse Transcription Kit (Thermo Fisher Scientifc, Inc., Waltham, MA, USA) was applied to obtain cDNA. The qRT-PCR was performed by using AceQ qPCR SYBR Green Master Mix (Vazyme, Nanjing, China). GAPDH was used as a reference control.

**Table:** 

Primer	Sequence (5′–3′)
ALPK2 primer-F	TCCGAAGGACCAGGGACTCTAT
ALPK2 primer-R	CGGTGAACCCCTTCTCCAAA
GAPDH primer-F	TGACTTCAACAGCGACACCCA
GAPDH primer-R	CACCCTGTTGCTGTAGCCAAA

### Western blot

First, HO-8910 and OVCAR-3 cells were collected and then lysed with cold RIPA lytic buffer (Cell Signal Technology, Danvers, MA, USA) containing protease inhibitors. BCA protein detection kit (HyClone-Pierce, Waltham, MA, USA, # 23225) was used to quantitatively extract protein. Western analysis was then performed using SDS-PAGE (10%). Blots were transferred to polyvinylidene difluoride (PVDF) membrane, incubated with 5% BSA and 0.5% Tween 20 for 1 h. It was important to note that antibody should be washed with TBST several times before and after addition. Then the primary antibodies (ALPK2, N-cadherin, Vimentin, Snail, Akt, p-Akt, Cyclin D1, CDK6, PIK3CA and GAPDH) were added and incubated overnight at 4 °C. Goat anti-rabbit IgG polyclonal antibody (1:3000) (Beyotime, Beijing, China, # A0208) labeled with horseradish peroxidase (HRP) was used for incubation for 1 h at room temperature. ECL-PLUS/Kit (Amersham, Chalfont, UK, # RPN2232) was used for color developing.

**Table:** 

Antibody name	Protein size (kDa)	Diluted multiples	Antibody source	Company	Number
ALPK2	237	1:1000	Rabbit	Abcam	ab111909
N-cadherin	125	1:1000	Rabbit	Abcam	ab18203
Vimentin	54	1:2000	Rabbit	Abcam	ab92547
Snail	29	1:1000	Rabbit	Abcam	3879S
Akt	60	1:1000	Rabbit	CST	4685
p-Akt	60	1:1000	Rabbit	Bioss	BS-5193R
Cyclin D1	36	1:2000	Rabbit	CST	2978
CDK6	37	1:1000	Rabbit	Abcam	ab151247
PIK3CA	110	1:1000	Rabbit	Abcam	ab40776
GAPDH	37	1:3000	Rabbit	Bioworld	AP0063

### MTT assay

We detected the proliferation of HO-8910 and OVCAR-3 cells by MTT assay. First, the cells were seeded to 96-well plates and incubated. After cells were digested by trypsin, complete medium was resuspended into cell suspension. The cell density was 2000 cells/well and inoculated to 96-well plates (100 µL/well) (Corning, Corning, NT, USA, #3599). MTT (3-(4, 5-dimethylthiazol-2-yl)-2, 5-diphenyl tetrazolium bromide) (Genview, Beijing, China; # JT343) solution was added per well. Afterwards, the medium with MTT was discarded and then dimethyl sulfoxide (DMSO) was added, shaking them for 10 min at room temperature. The absorbance was measured at 490 nm with Microplate Reader and the cell viability was calculated.

### Detection of cell cycle by fluorescence activated cells sorting (FACS)

Transfected HO-8910 and OVCAR-3 cells with shRNA lentivirus were cultured. After then, PBS containing 0.1% BSA was added, then the cell suspension was centrifuged at 200*g* for 5 min. Cells were fixed with ethanol, then stained by propidium iodide (PI). Distribution ratio of G1, S and G2 cell cycle of the ALPK2 knockdown group and the control group were detected and analyzed by flow cytometry.

### Flow cytometry apoptotic assay

HO-8910 and OVCAR-3 cells in ALPK2 knockdown group and control group were incubated in 6 cm culture dish for 5 days, which was digested with trypsin and resuspended. Annexin V-APC was added and stained in dark for 15 min. Depending on the number of cells, 800 μL of binding buffer is added and samples are tested on the machine. Analysis was performed using flow cytometry software guava InCyte.

### Wound-healing assay

Approximately 3 × 10^4^ transfected cells were added into the hole, and the confluence of cells reached more than 90%. The next day, the low concentration serum medium (e.g., 0.5% FBS) was changed and the scratch instrument was used to aim at the central part of the lower end of the 96-well plate and push it up gently to form a scratch. After gently rinsing with serum-free medium 3 times, low concentration serum medium was added and photographed.

### Human apoptosis antibody array

Human apoptotic antibody array kit (# AB134001) was applied to detected proteins related to apoptotic signalng pathway. Cell samples were collected after lentivirus transfection, washed with PBS, lysed for 30 min, and gently shaken well. The extracted total protein was diluted to 0.5 mg/mL with the array dilution buffer kit. Each antibody array membrane was sealed with a sealed buffer at room temperature for 30 min and gently shaken overnight. HRP linked Streptavidin was added to the membranes. Protein was visualized using ChemiDoc XRS chemiluminescence detection and imaging system. The density of the spots was quantitated using Quantity One software and normalized to the *α*–tubulin levels.

### Animal xenograft model

The animal experiment was approved by the Ethics Committee of The IRB of Third Xiangya Hospital, Central South University. BALB/c female nude mice (4 weeks old) were purchased from Shanghai Jiesijie Experimental Animals Co., Ltd (Shanghai, China). HO-8910 cells transfected with shALPK2 or shCtrl were subcutaneously injected into BALB female nude mice (4 × 10^6^ cell per mouse). Data were collected after 7 days of injection of HO-8910 cells, and then tumor volume and weight of the mice were measured daily for 21 days. Subsequently, tumor load was assessed using bioluminescence imaging and IVIS spectral imaging system (emission wavelength 510 nm). After 21 days, the mice were sacrificed by injection of sodium pentobarbital, and the tumors were removed and collected. Finally, the tumor was weighed and photographed.

### Ki-67 staining

Tumor tissues were sectioned from the sacrificed mice. Afterwards, they were repaired and blocked with the citrate antigen. Antibody Ki67 (1: 200, Abcam, USA, Cat. # ab16667) was added to the shALPK2 or shCtrl, respectively. Subsequently, they were mixed and incubated overnight. PBS elution was performed several times before and after antibody addition. Secondary antibody IgG (1: 400, Abcam, USA, Cat. # ab6721) was added and incubated at room temperature for 30 min. Tissue slices were first stained with DAB, and then with hematoxylin. Images were collected with a photomicroscope and analyzed.

### Statistical analysis

The data were expressed as mean ± Standard Error Mean (SEM), (n ≥ 3) and pairwise comparisons were analyzed by Student’s t-test; *P* values less than 0.05 were considered statistically significant. All the statistics and graphs were performed and analyzed with GraphPad Prism 6 software (GraphPad Software Inc., San Diego, CA, USA). The qRT-PCR was analyzed by 2^−∆∆CT^ method. T-test were used to compare the difference.

## Results

### ALPK2 is abundantly expressed in OC tissues

**Fig. 1:**
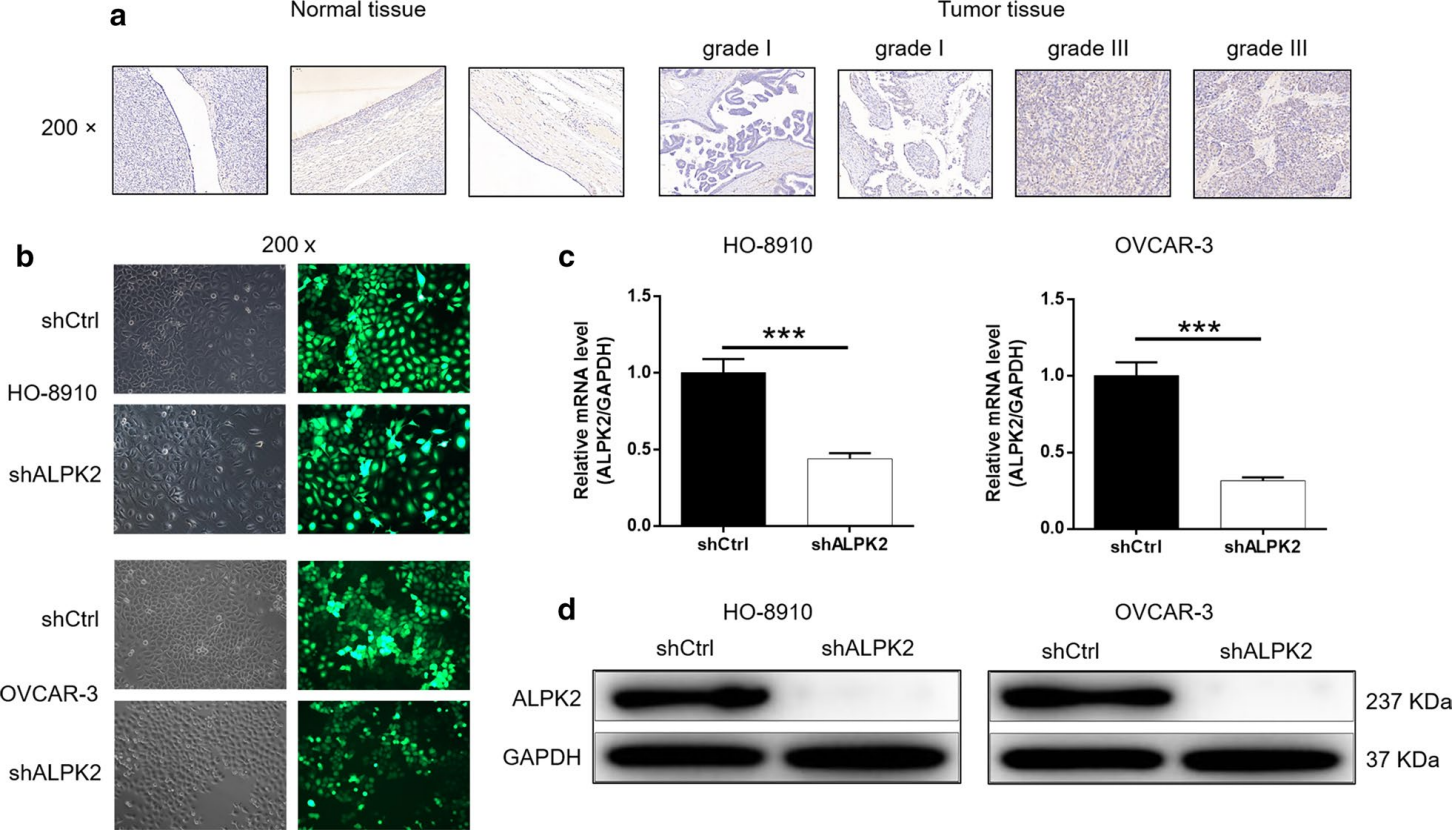
ALPK2 is highly expressed in OC tissues and the construction of ALPK2 knockdown cell model. **a** The expression of ALPK2 in the normal and tumor samples detected by IHC. **b** Transfection efficiency for HO-8910 and OVCAR-3 cells was evaluated by expression of green fluorescent protein 72 h post-infection. **c**, **d** The specificity and validity of the lentivirus-mediated shRNA knockdown of ALPK2 expression was verified by qRT-PCR (**c**) and Western blot (**d**). The data were presented as the mean ± SD (n = 3), *P < 0.05, **P < 0.01, ***P < 0.001

Based on IHC analysis, the expression of ALPK2 in OC tissues was significantly higher than that in normal ovarian tissues (*P *< 0.001) (Table 1) (Fig. 1a). Furthermore, we observed that significant correlation between ALPK2 expression and pathological grade of OC through performing Mann–Whitney U analysis (Table 2). Further combined with Spearman rand correlation analysis, the expression of ALPK2 was positively correlated with pathological grade (Table 3). In summary, ALPK2 may be related to the development of OC.

**Table 1:** Expression patterns in ovarian cancer tissues and normal tissues revealed in immunohistochemistry analysis

ALPK2 expression	Tumor tissue		Normal tissue		*P* value
	Cases	Percentage (%)	Cases	Percentage (%)	
Low	79	42	66	100	< 0.001
High	109	58	0	–

**Table 2:** Relationship between ALPK2 expression and tumor characteristics in patients with ovarian cancer

Features	No. of patients	ALPK2 expression		*P* value
		Low	High	
All patients	188	79	109	
Age (years)				0.728
< 49	90	39	51	
≥ 49	98	40	58	
Grade				< 0.001
1	23	19	4	
2	33	28	5	
3	113	19	94	
Stage				0.499
I	144	56	88	
II	14	7	7	
III	12	6	6	
IV	2	0	2	
T infiltrate				0.450
T1	144	56	88	
T2	22	10	12	
T3	6	3	3	
Lymphatic metastasis (N)				0.710
N0	161	64	97	
N1	11	5	6

**Table 3:** Relationship between ALPK2 expression and tumor characteristics in patients with ovarian cancer

ALPK2		*P* value
Grade	Pearson correlation	0.632
	Significance (double tail)	< 0.001
	N	169

### Construction of ALPK2 knockdown cell models

After 72 h of incubation, the cell transfection efficiency was more than 80% and the cell state was normal under fluorescence microscope (Fig. 1b). Moreover, qRT-PCR showed that the expression of ALPK2 in shALPK2 group was downregulated by more than 55% compared with shCtrl group in both OC cell lines (*P *< 0.001) (Fig. 1c). Similar trend was also observed in Western blot analysis (Fig. 1d). All the above results indicated that the ALPK2 knockdown cell model was successfully constructed.

### Silencing of ALPK2 inhibits cell proliferation

**Fig. 2:**
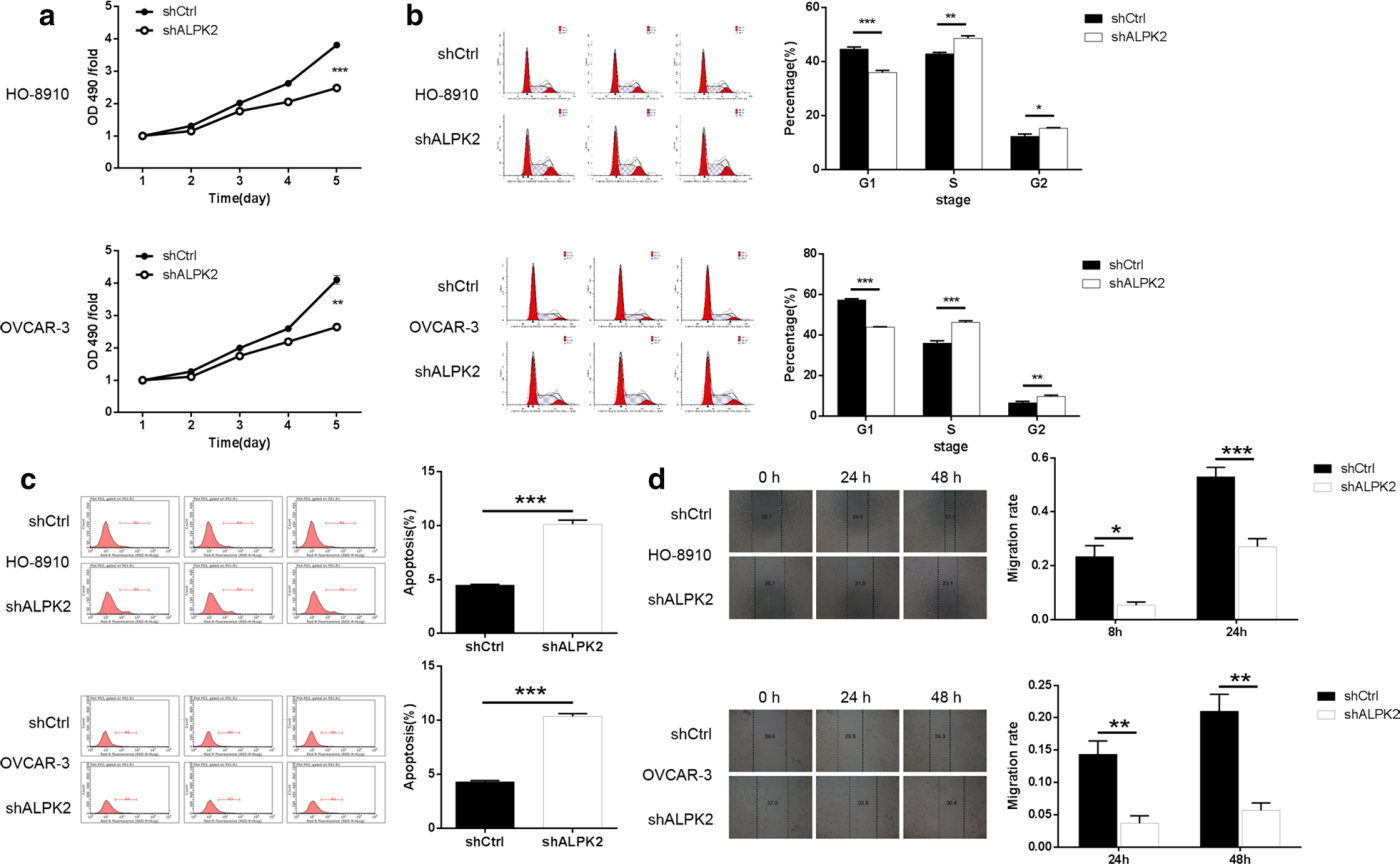
Knockdown of ALPK2 inhibits cell proliferation, cell cycle and migration, promotes apoptosis in OC cells. **a** Cell proliferation of HO-8910 and OVCAR-3 cells with or without knockdown of ALPK2 was evaluated by MTT assay. **b** The HO-8910 and OVCAR-3 cells in the G1, S and G2 phases of the ALPK2 gene knockdown group and the control group were detected by flow cytometry. **c** Flow cytometry analysis based on Annexin V-APC staining was utilized to detect the percentage of early apoptotic cell for HO-8910 and OVCAR-3 cells. The X axis indicated the cell apoptosis while the Y axis indicated the green fluorescence detected from the GFP tagged on lentivirus (shALPK2 or shCtrl). **d** Cell migration of HO-8910 and OVCAR-3 cells with or without knockdown of ALPK2 was evaluated by wound-healing assay. The data were expressed as mean ± SD (n = 3), *P < 0.05, **P < 0.01, ***P < 0.001

HO-8910 and OVCAR-3 cells transfected with shALPK2 or shCtrl were cultured for 5 days, and the effects of ALPK2 on OC cell growth were detected by MTT assay. As shown in Fig. 2a, the results of MTT assay displayed that cell proliferation of HO-8910 cell lines were suppressed notably by the knockdown of ALPK2 (*P *< 0.001). At the same time, the proliferation ability of OVCAR-3 cell lines were moderately inhibited after the knockdown of ALPK2 (*P *< 0.01). These results suggested that ALPK2 exerts an important role in proliferation of OC cells.

### Silencing of ALPK2 arrests cell cycle and induces apoptosis of OC cells

To examine the effects of ALPK2 on cell cycle and apoptosis of OC cells, Annexin V staining was used for flow cytometry analysis. Cell cycle was detected by flow cytometry in both cell lines. As a result, as shown in Fig. 2b, the percentage of G2 cells in HO-8910 and OVCAR-3 cells increased significantly as compared to the shCtrl group (*P* < 0.001). As shown in Fig. 2c, the percentage of apoptosis in HO-8910 and OVCAR-3 cells increased by almost threefolds as compared to the shCtrl group (*P *< 0.001). Collectively, the silencing of ALPK2 significantly arrested cell cycle and promoted cell apoptosis in OC cells.

### Silencing of ALPK2 inhibits migration of OC cells

Metastasis is an important factor that limits the curative effect of chemotherapy for OC treatment. Therefore, in a bid to investigate the effect of ALPK2 on the metastasis of OC, the wound-healing assay was performed to evaluate the migration ability of OC cells. Obviously, the migration of HO-8910 and OVCAR-3 cells was inhibited by at least 49% and 76%, respectively (*P *< 0.001) (Fig. 2d). Consequently, it might be concluded that ALPK2 also plays a vital role in the metastasis of OC.

### Silencing of ALPK2 downregulated expression of EMT protein

**Fig. 3:**
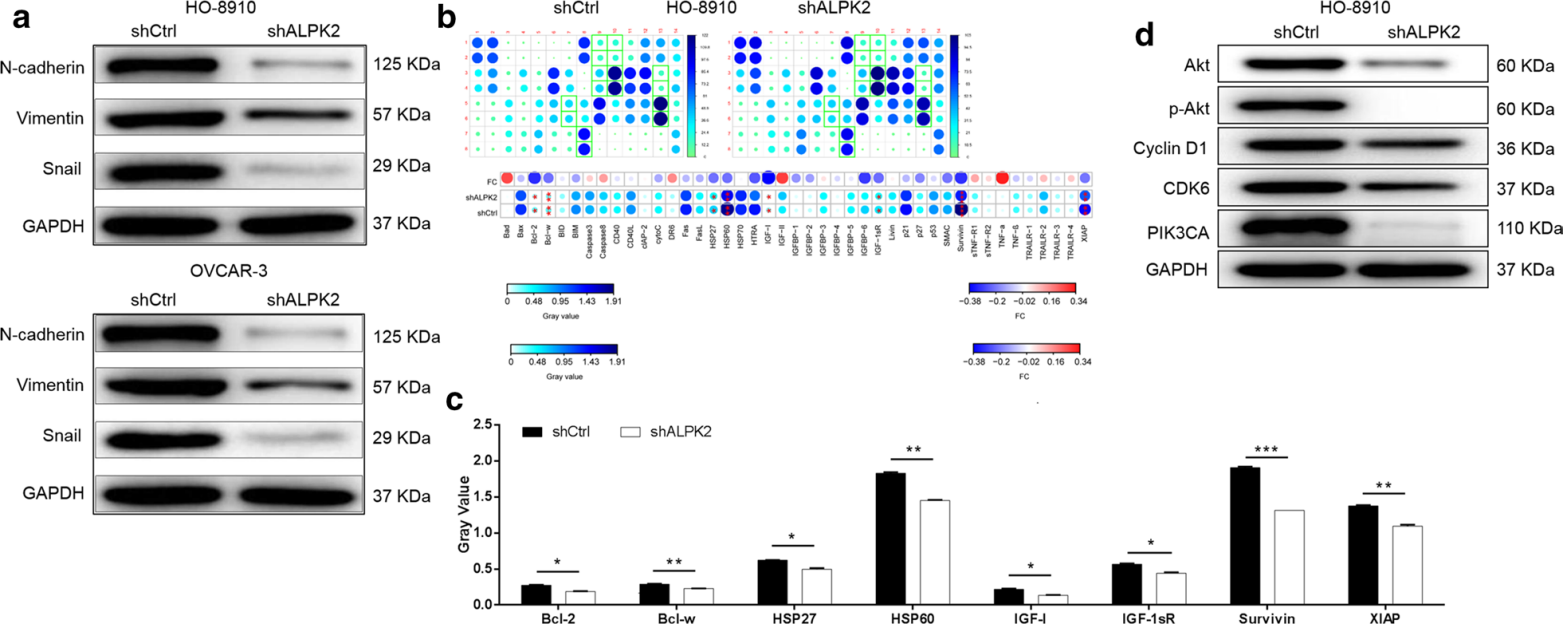
Exploration of downstream molecular mechanism of ALPK2 in OC cells. **a** Human apoptosis antibody array analysis was performed in HO-8910 cells with or without ALPK2 knockdown. **b** Densitometry analysis was performed and the gray values of differentially expressed proteins were shown. **c** The expression of epithelial-mesenchymal transition (EMT) proteins were observed by Western blot in HO-8910 and OVCAR-3 cells. **d** The expression of target proteins pathways were observed by Western blot in HO-8910 cells. The data were expressed as mean ± SD (n = 3), *P < 0.05, **P < 0.01, ***P < 0.001

In order to detect the effect of ALPK2 on the expression of Epithelial-Mesenchymal Transition (EMT) protein in OC cells, Western blot was performed. It is not difficult to found that the protein levels of N-cadherin, Vimentin and Snail in the shALPK2 group were downregulated compared with those in the shCtrl group in the cells of HO-8910 and OVCAR-3 (Fig. 3a). Based on these findings, we may conclude that knockdown of ALPK2 activate EMT.

### Exploration of downstream molecular mechanism of ALPK2 in OC cells

To explore the potential mechanism of the regulatory ability of ALPK2 knockdown in OC, human apoptotic antibody arrays were used to analyze the differential expression of 43 proteins between HO-8910 cells in shALPK2 and shCtrl groups. After silencing ALPK2 in HO-8910 cells, the expression levels of Bcl-2, Bcl-w, HSP27, HSP60, IGF-I, IGF-1sR, Survivin and XIAP were dramatically downregulated (*P *< 0.05) (Fig. 3b, c). Apparently, the results were consistent with the aforementioned cellular experiments especially the cell apoptosis assay. Moreover, Western blot was also applied to detect the expression of proteins in downstream signaling pathways in HO-8910 cells. Compared with the negative group, protein expression of Akt, p-Akt, Cyclin D1, CDK6 and PIK3CA were downregulated in group shALPK2 (Fig. 3d).

### Silencing of ALPK2 in OC cells impaired tumorigenesis in vivo

**Fig. 4:**
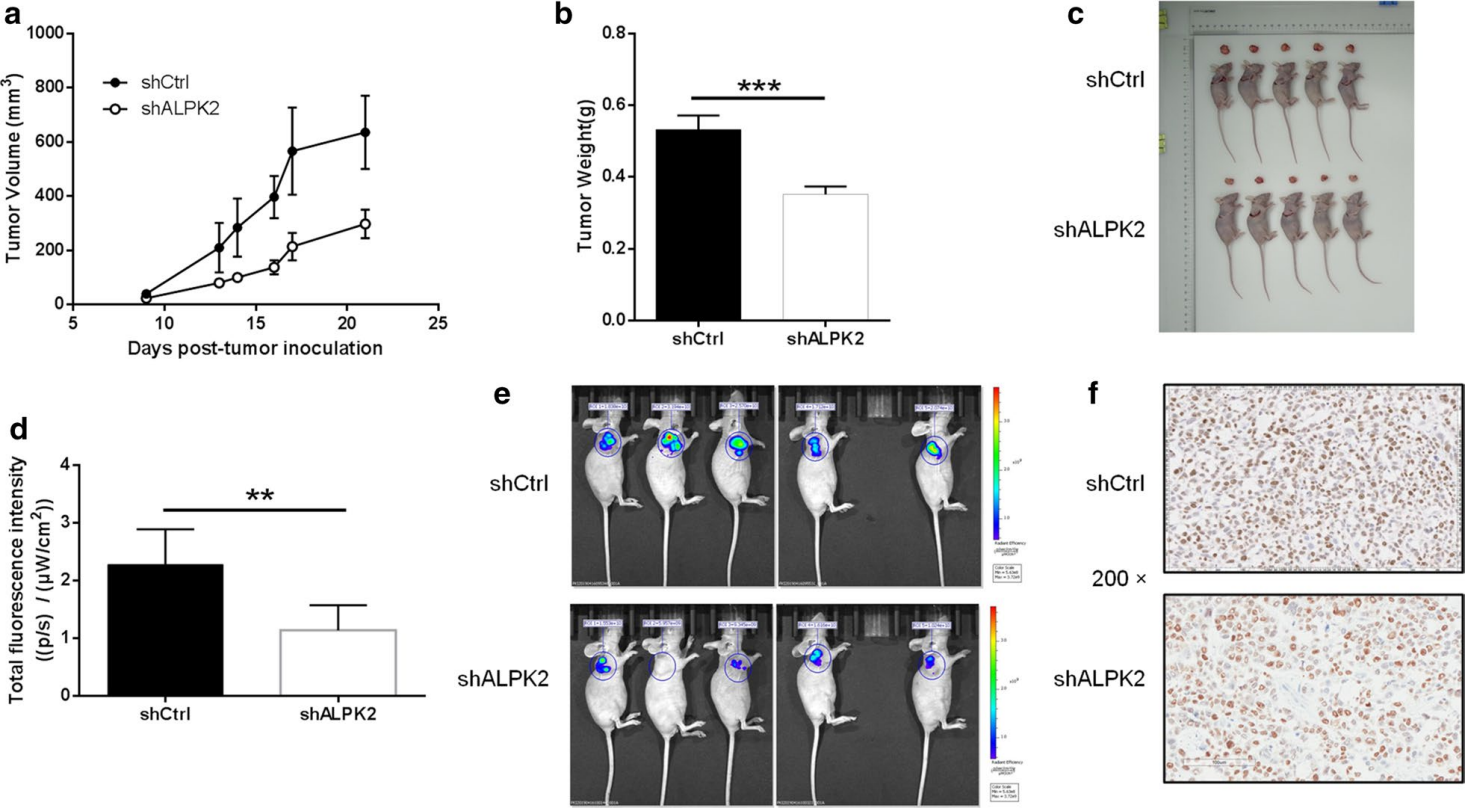
Knockdown of ALPK2 inhibits tumor growth in mice xenograft models. **a** The volume of tumors in shCtrl group and shALPK2 group was measured post-injection. **b** The average weight of tumors in shCtrl group and shALPK2 group. **c** The photos of tumors in shCtrl group and shALPK2 group. **d** The total bioluminescent intensity of tumors in shCtrl group and shALPK2 group. **e** The bioluminescence imaging of tumors in shCtrl group and shALPK2 group. **f** Images of the tumor tissues of the shCtrl group and shALPK2 group with Ki-67 staining. The data were expressed as mean ± SD (n = 3), *P < 0.05, **P < 0.01, ***P < 0.001

The above assay has confirmed that ALPK2 could promote cell proliferation and migration, while inhibiting cell apoptosis in vitro. We still wondered whether silencing of ALPK2 affects OC growth in vivo. As a consequence, HO-8910 cells were subcutaneously injected into nude mice and a mouse xenograft model was established to verify this issue. As shown in Fig. 4a, the average volume of tumor of shALPK2 group was sharply decreased in comparison with that of the shCtrl group (*P* < 0.01). The average tumor weight of mice model in shALPK2 group was also markedly lower 180 ± 18 mg than that shCtrl group (*P *< 0.05) (Fig. 4b, c). What’s more, bioluminescence imaging suggested that the intensity of bioluminescence markedly decreased in shALPK2 group than that in shCtrl group (*P *< 0.05) (Fig. 4d, e). In addition, Ki-67 staining also displayed that the proliferation index of tumor tissue in shALPK2 group was significantly lower than that in negative group (Fig. 4f). To sum up, these results suggested that the tumorigenicity of ALPK2 is attenuated in vivo, which is consistent with the above data in vitro.

## Discussion

OC is a significantly heterogeneous malignant tumor characterized by various histological subtypes. More than 90% of OC have the characteristics of unstable gene, strong invasiveness and poor clinical curative effect [14, 17, 18]. The inadequacy of existing treatment methods suggested that we need to thoroughly understand the molecular mechanism of OC in order to find more optional molecular targets. Xi et al. indicated that the high expression of CHAF1A affected the growth of OC cells, which might be a biomarker for prognosis and a new molecular target for OC [19]. In addition, Katherine et al. found that collagen receptor DDR2 plays a key role in OC metastasis and is considered to be an important breakthrough in controlling OC metastasis [20]. Despite these results, the underlying mechanism for the development and metastasis of OC remains unclear. Therefore, more effective therapeutic strategies, especially molecular targeting agents, are needed to improve the survival rate of patients with advanced OC.

At present, research on the mechanism of ALPK2 in cancer is still scarce. For example, Nishi et al. believed that mutations in the ALPK2 disrupt the location of filamentous actin in colorectal surface cells [21]. In addition, ALPK2 has been found to promote the development of human pluripotent stem cells in the heart [22]. Salim et al. demonstrated that ALPK2 is regulated by miRNA-214 and has been linked to invasive human non-small cell lung cancer [23]. Other studies found that ALPK2 is associated with the prognosis of gastric cancer [24]. These findings prompted us to explore the effects of ALPK2 in OC cells.

In this study we first clarified that ALPK2 expression is upregulated in OC, which involved in proliferation, apoptosis, cycle distribution and migration of OC cells. Furthermore, ALPK2 was involved in OC cells via regulating EMT-related proteins. The association of EMT with ovarian carcinogenesis and progression was also demonstrated by other studies. During EMT, cancer cells lose epithelial characteristics and acquire mesenchymal properties that promote extracellular matrix invasion and distant metastasis [25]. This process involves a variety of molecular mechanisms, such as the downregulation of E-cadherin, the acquisition of the transcription factor Snail, N-cadherin and Vimentin [26, 27]. What’s more, we believed that ALPK2 affects the OC cells via activation of EMT, represented by the altered expression of N-cadherin, Vimentin and Snail.

The initiation of apoptosis occurs through strictly controlled mechanisms. The decrease of apoptosis is an important factor in tumorigenesis and carcinogenesis [28]. Bcl-2 has certain effects on apoptosis of OC cells [29], which attenuates cytotoxicity by downregulating mitochondrial Ca^2+^ signaling, may be a potential therapeutic target for OC [30]. In addition, Zhao et al. demonstrated that the serum level of HSP27 in OC is correlated with peritoneal metastasis, which can be used as a potential additional indicator to determine epithelial peritoneal metastasis [31]. The stable and protein translation of the mitochondrial protein regulated by HSP60 promotes the growth of OC proved by Guo et al. [32]. IGF system plays an important role in the occurrence, maintenance, progression, survival and chemotherapy response of OC [33]. Liefers-Visser et al. holds this view that Survivin is closely related to OC stage and tumor grade, which may be a new clinicopathological marker of OC [34]. On the other hand, high expression of XIAP is associated with paclitaxel resistance in OC, reducing its expression to promote apoptosis and increasing the sensitivity of drug-resistant cancer cells to paclitaxel [35]. Therefore, silencing ALPK2 participates in OC cell apoptosis by initiating a series of apoptosis-related proteins, such as Bcl-2, Bcl-w, HSP27, HSP60, IGF-1, IGF-1sR, Survivin, and XIAP.

Besides, we have shown that OC cells can affect downstream signaling pathways after silencing ALPK2. In the past few years, there are accumulated quantity of coverage about the involved signaling pathway in OC. PIK3CA is a catalytic subunit of PI3K, which is activated in ovarian clear cell carcinoma [36]. It has been reported recently that PI3K/Akt/mTOR pathway is often mutated or activated in OC and plays an important role in its development. Inhibition of this pathway may be effective in the treatment of OC [37]. Cyclin D1 overexpression was observed in some OC cases, which could be used as an independent prognostic indicator for overall survival of patients [38, 39]. Moreover, silencing of Cyclin D1 blocks the G0/G1 phase of OC cells [40]. Dall’ Acqua et al. clearly supported that inhibition of CDK6 induces OC cell death. Indeed, CDK4/6 inhibitors have been successfully applied in cancer patients to improve the prognosis of OC patients [41]. Herein, we concluded that ALPK2 is involved in OC cells along with the regulation of downstream protein pathway (Akt, p-Akt, Cyclin D1, CDK6 and PIK3CA). Combined with this study and previous conclusions, it is not difficult to find that the regulation network of ALPK2 in OC is a complex system, and efforts should be made to further clarify its regulation mechanism.

## Conclusion

In conclusion, this study confirmed for the first time that the upregulation of ALPK2 expression in OC, which can promote the proliferation, migration, arrest cell cycle, and suppress apoptosis of OC cells. In addition, it was proved that the knockdown of ALPK2 can participate in OC cell migration and apoptosis by activating EMT, regulating the expression of apoptosis-related proteins and downstream signaling pathways. This study has made some contributions to the pathogenesis of OC, so we can infer that ALPK2 may be an effective target for treating OC.

## Data Availability

The datasets used and/or analysed during the current study are available from the corresponding author on reasonable request.

## Abbreviations

ALPK2: Alpha protein kinase 2
OC: Ovarian cancer
IHC: Immunohistochemical staining
PVDF: Polyvinylidene difluoride
EMT: Epithelial-Mesenchymal Transition
